# Flavonoid-Inspired Vascular Disrupting Agents: Exploring Flavone-8-Acetic Acid and Derivatives in the New Century

**DOI:** 10.3390/molecules26144228

**Published:** 2021-07-12

**Authors:** Silvia Gobbi, Federica Belluti, Angela Rampa, Alessandra Bisi

**Affiliations:** Department of Pharmacy and Biotechnology, Alma Mater Studiorum-University of Bologna, Via Belmeloro 6, I-40126 Bologna, Italy; federica.belluti@unibo.it (F.B.); angela.rampa@unibo.it (A.R.); alessandra.bisi@unibo.it (A.B.)

**Keywords:** angiogenesis, VDAs, natural products, flavone-8-acetic acid, DMXAA, vadimezan

## Abstract

Naturally occurring flavonoids are found as secondary metabolites in a wide number of plants exploited for both medicine and food and have long been known to be endowed with multiple biological activities, making them useful tools for the treatment of different pathologies. Due to the versatility of the scaffolds and the vast possibilities of appropriate decoration, they have also been regarded as fruitful sources of lead compounds and excellent chemical platforms for the development of bioactive synthetic compounds. Flavone-8-acetic acid (FAA) and 5,6-dimethylxanthone acetic acid (DMXAA) emerged for their antitumour potential due to the induction of cytokines and consequent rapid haemorrhagic necrosis of murine tumour vasculature, and different series of derivatives have been designed thereafter. Although the promising DMXAA failed in phase III clinical trials because of strict species-specificity, a boost in research came from the recent identification of the stimulator of interferon genes (STING), responsible for supporting tumoural innate immune responses, as a possible biological target. Consequently, in the last decade a renewal of interest for these flavonoid-based structures was noticed, and novel derivatives have been synthesised and evaluated for a deeper understanding of the molecular features needed for affecting human cells. Undoubtedly, these natural-derived molecules deserve further investigation and still appear attractive in an anticancer perspective.

## 1. Introduction

The term angiogenesis refers to the development of new capillaries from existing blood vessels, and it plays a pivotal role in different physio-pathological processes, being involved in embryo development, in wound healing but also in various diseases. In particular, when dysregulated, angiogenesis has a major impact on human health, becoming involved in the onset of ischemic, inflammatory and malignant disorders [1]. In normal physiological conditions, angiogenesis is regulated by a tight network of growth factors and cytokines, including vascular endothelial growth factor (VEGF), fibroblast growth factors (FGFs), tumour necrosis factor alpha (TNF-α), epidermal growth factor (EGF), interleukin 8 (IL-8) and erythropoietin (EPO), produced by various types of cells, which include endothelial, smooth muscle, inflammatory and cancer cells [2].

Undoubtedly, the fast proliferation of cancer cells requires constant feeding, ensured by a continuous blood flow into the tumour microenvironment. Therefore, one of the key steps in cancer progression and formation of metastases is the development of new blood vessels, endowed with peculiar features with respect to normal vascular tissue. Indeed, tumour microvasculature is usually defined as immature, characterized by poorly structured walls, layered by irregularly shaped endothelial cells, defectively connected with pericytes and endowed with a high intrinsic permeability to macromolecules, consistent with the imbalance between pro-angiogenic factors, such as VEGF, and anti-angiogenic ones. It has been widely reported that tumours are unable to properly respond to vasoactive agents acting on specific vascular smooth muscle cell receptors, mainly due to the deficiency of vascular smooth muscle cells [3]. The heterogeneity of blood flow, together with the unusually elevated levels of deoxygenated blood, are to be held primarily responsible for chronic and cycling hypoxia, decreased cellular pH and increased interstitial pressure found in tumours. Hypoxia, in turn, through the activation of hypoxia-inducible factor (HIF), activates pro-angiogenic factors, among which is VEGF itself [4]. This abnormal vascular network impairs the ability of conventional anticancer drugs to enter cancer cells, reducing their therapeutic efficacy, and promotes cancer metastasis, drug resistance progression and immune evasion. Moreover, recent studies suggest that vascular homeostasis could be directly affected by a series of individual differences, related to genetic polymorphisms, specific mutations and individual cancer types, paving the way for personalized medicine, thus enabling the selection of the best therapeutic treatment according to the peculiar variability of tumours [5]. Therefore, it is not surprising that angiogenesis is still considered a promising and effective target in anti-cancer therapy.

Currently, two strategies are followed for attaining this goal: anti-angiogenic therapy, aimed at inhibiting new blood vessels formation with anti-angiogenic agents (AAs) and anti-vascular therapy, in which preformed tumour blood vessels are destroyed by means of vascular disrupting agents (VDAs). The anti-angiogenic therapy acts by significantly reducing cancer cells nutrient and oxygen supply. AAs belongs to different main groups, among which small molecule inhibitors of angiogenesis, targeting different pathways involved in the process, and monoclonal antibodies are the prevailing ones for cancer treatment. The main drawbacks for clinical use of AAs remain the off-target effects of this drugs in healthy tissues and the development of drug resistance [2]. On the other hand, VDAs can be grouped into three subclasses, namely microtubule destabilizing agents, anti-vascular-acting flavonoids and drugs directly targeting endothelial cells, all causing destruction of tumour vasculature through apoptosis or necrosis of endothelial cells. Comparing the antitumour profile of classic anticancer drugs acting on tumour cells and compounds targeting tumour blood vessels, VDAs appear the most promising, mainly due to their low induction of resistance, and their ability to reach the target blood vessels and to induce substantial necrosis of the tumour core [6]. Unfortunately, one of the main disadvantages associated with VDAs is the incomplete eradication of the tumour, due to the induction of a central necrosis that is surrounded by preserved vascularized cells, able to survive and proliferate. This so-called “narrow rim” of peripheral cancer cells could then induce rapid tumour regrowth. Accordingly, most VDA-based therapeutic approaches still entail combination treatments, acting with multiple mechanisms and able to both inhibit tumour growth and its vasculature [7]. Notably, it is of pivotal importance to follow a proper combined treatment schedule, considering that the reduced blood flow and the altered permeability of blood vessels induced by VDAs in the tumour environment may induce a deep modification in the distribution of simultaneously administered chemotherapeutic agent.

**Figure 1:**
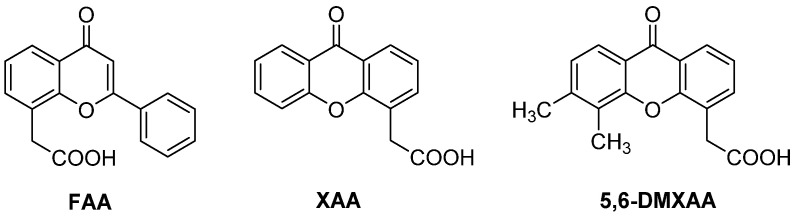
Structures of prototypes flavone-8-acetic acid (FAA), xanthone-4-acetic acid (XAA) and 5,6-dimethylxanthone-4-acetic acid (DMXAA).

In recent years, an increasing number of natural products (NPs) or NP-derived compounds appeared on the scene as VDAs, mainly acting as microtubule destabilizers or apoptosis inducers selectively on cancer endothelial cells. In particular, combretastatins, stilbene derivatives extracted from the bark of the *Combretum caffrum* tree, are the most known VDAs in the microtubule destabilizing group. These compounds act by binding to the colchicine binding site of tubulin in endothelial cells, inhibiting tubulin polymerization and inducing G2/M cell cycle arrest. The resulting activation of the apoptosis machinery leads to endothelial cells death, blood vessels disruption and finally tumour necrosis. A number of combretastatin-based derivatives were submitted to clinical trials, both as single agents and in combination with other anticancer drugs, giving better clinical outcomes when administrated as combinations. On the other hand, flavonoids, plant secondary metabolites found in food, mainly vegetables, fruits and cereals, showed a myriad of beneficial properties, among which were antioxidant, anti-inflammatory, anti-mutagenic and anti-carcinogenic [6,7] properties, and different mechanisms of action were proposed to elucidate their activity in several stages of cancer progression. In particular, they are known to play a relevant role as VDAs by inducing apoptosis. The flavonoid core structure can also be regarded as a validated synthetic platform, which upon appropriate functionalisation can provide a huge number of structurally-related compounds endowed with distinct biological profiles. In particular, flavonoid-related compounds have been screened in clinical trials for cancer treatment, leading in 1985 to the identification of flavone acetic acid (FAA, Figure 1) [8], acting by promoting haemorrhagic necrosis of subcutaneous murine colon tumours. Following experiments indicated that FAA might induce a cytotoxic effect due to its ability to selectively impair tumour blood flow. Although this compound failed in clinical studies in humans, this result stimulated the synthesis of many FAA analogues, aimed at better clarifying the mechanism of action and at overcoming the observed species specificity issue. The tricyclic analogue of FAA, xanthenone-4-acetic acid (XAA, Figure 1) [9], proved to be as active as FAA and suitable for structural modifications, which allowed the structure activity relationships (SARs) for this promising class of VDAs to expand. With the discovery of the highly potent 5,6-dimethylxanthenone-4-acetic acid (DMXAA, ASA404, Vadimezan, Figure 1) [10,11], a safe and well-tolerated VDA targeting immature and unstable vasculature of solid tumours [7], the goal seemed to have been reached.

A dual mechanism of antitumour effect has been proposed for DMXAA. A direct effect is related to its ability to induce apoptosis in tumour endothelial cells, with concurrent haemorrhagic necrosis in tumour tissues [12]. This outcome appears shortly after administration and is followed by the activation of the innate immune system, representing the indirect effect of DMXAA, not usually found in other VDAs. This activation leads to inflammatory cytokine production and tumour-specific inflammatory responses. Once more, despite the exciting antitumour effects observed in mouse models, DMXAA also failed when combined with standard-of-care chemotherapy in a phase III human efficacy trial. In 2012, further mechanistic studies led to the identification of the possible target of this compound, the stimulator of interferon genes (STING, also known as ERIS, MITA, TMEM173 and MPYS), an endoplasmic reticulum adaptor transmembrane protein involved in the innate immune pathway [13]. Recently, the pivotal role of STING in sustaining innate immune responses in the tumour microenvironment has been validated [14]; and structure–function studies of mouse STING (mSTING) and human STING (hSTING) suggested that DMXAA is a potent agonist of mSTING, while polymorphism in hSTING makes it unable to bind the compound, and this probably explains its failure in clinical trials [15]. Taken together, all these findings provide a mechanistic insight for the lack of efficacy of DMXAA in human tumours and give the rationale for carrying out further SAR studies on the structures of FAA and XAA, in order to overcome this drawback and obtain effective new VDAs.

This review aims to give an overview of the last 20 years of medicinal chemistry studies on FAA and XAA, endowed with a peculiar and intriguing antitumour profile, involving both vascular disrupting activity and immunomodulation. The first decade marks the end of intense years of medicinal chemistry efforts, leading to a plethora of derivatives with no major improvement in anticancer activity. In the last decade, the revived interest in these flavonoid-related molecules allowed the tangle of the targets involved in their action to be partially unravelled and for more promising compounds to be identified, in part due to more focused biological testing.

## 2. SAR Studies 2001–2009

Different investigations on FAA and XAA were performed in the last 20 years, and our research group was deeply involved in establishing appropriate SARs for the two related compounds aimed at identifying an effective VDA and gaining more insight into the peculiar mechanism of action of these drugs. Indeed, the biological targets involved were still unknown, and studies mainly focused on the definition of structural requirements for both immune modulation and induction of cytokines such as TNFα, also involved in vascular disrupting properties of the parent compounds [6], to gain enhanced anticancer potential.

### 2.1. FAA Analogues

**Figure 2:**
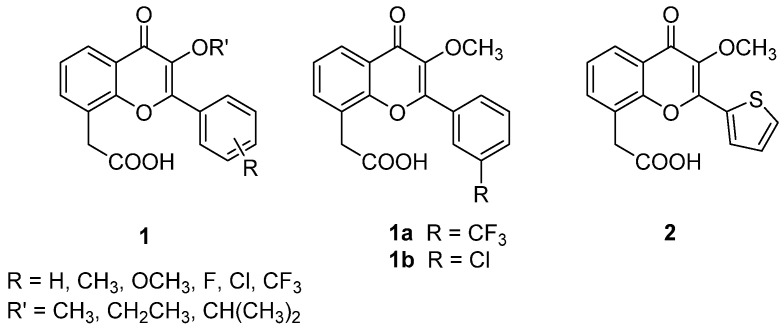
3-Alkoxy FAA derivatives.

Continuing our previous studies and considering the limited SARs available to date for the lead FAA, in 2003 new analogues were synthesized, in which an alkoxy group (mainly methoxy, then modified to ethoxy and isopropoxy) was inserted in position 3 and a functionalised benzene ring or a furan moiety were introduced in position 2 [16]. The compounds (**1** and **2**, Figure 2) were tested to evaluate both their direct cytotoxicity on different adenocarcinoma cell lines and their ability to induce an indirect toxic effect mediated by murine macrophages and human monocytes co-cultured with them.

All compounds were endowed with very low direct cytotoxicity, but most of them enhanced the antitumour effect of immune cells, showing remarkable activity on human monocytes, which was not observed before with FAA derivatives, and a certain degree of species specificity could also be noted among the different compounds. Notably, activity was lost with the introduction of a methoxy group in position 3 of the flavone core, while compounds bearing ethoxy or isopropoxy functions showed activity on murine macrophages, but not on human monocytes. The introduction of substituents on the 2-phenyl ring allowed activity to be restored, even if neither their position nor their chemical properties seemed to play a significant role. The most interesting compounds proved to be **1a** and **1b** (Figure 2), carrying electron-withdrawing groups in position 3′ more potent than the reference compound. In particular, **1a** showed higher activity than DMXAA in the human model, while it had no effect on murine macrophages. The two compounds also proved to induce TNF-α production by human peripheral blood mononuclear cells (HPBMCs).

**Figure 3:**
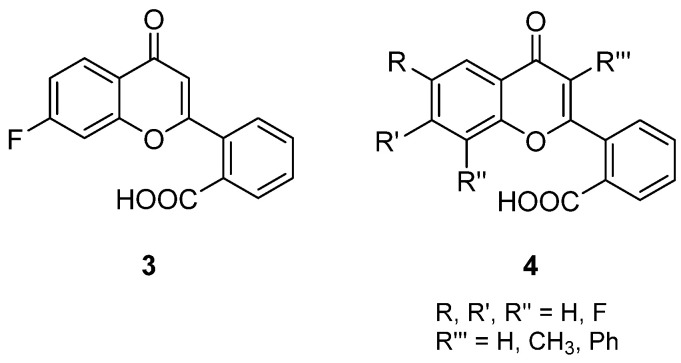
Fluorinated FAA derivatives.

As a follow-up of a previous study in which the carboxy function of FAA was moved to position 2′ on the 2-benzene ring, thus maintaining the spatial relationship with the flavone core, and the most interesting compound carried a fluorine atom in position 7 (**3**, Figure 3) [17], in a subsequent paper the synthesis of other mono- or difluorinated flavones (**4**, Figure 3) [18] was reported.

Direct cytotoxicity for the new compounds was seen only at very high concentrations. When tested for the potential enhancement of antitumour activity mediated by macrophages and human monocytes, it was seen that the introduction of fluorine atoms in the flavone nucleus led to an increase in activity of murine macrophages, while no effect was produced in human models with any of the new derivatives, showing a similar profile to the parent compound FAA.

**Figure 4:**
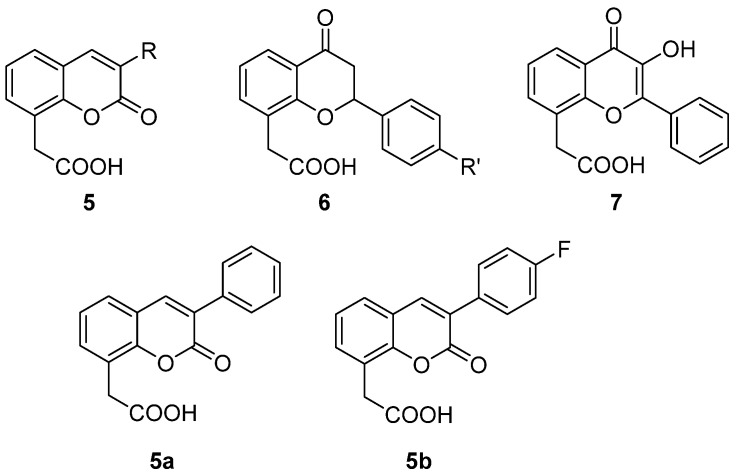
Naturally-inspired coumarin-, flavanone- and flavonol-based FAA derivatives.

In a more recent study, some previously synthesized naturally-inspired coumarin, flavanone and flavonol derivatives structurally-related to FAA (**5**, **6**, **7**, Figure 4) and for which cytotoxic activity on a human adenocarcinoma cell line was described [19], were reconsidered for further evaluation. In particular, the potential to activate the immune system by inducing lytic properties, TNF-α and nitric oxide in a cell line of human monocytes (Mono Mac 6, MM6) was investigated [20].

Despite the very low cytotoxicity observed, some compounds showed a remarkable indirect toxic effect on tumour cells co-cultured with human MM6 cells, comparable to DMXAA for the coumarin-based **5a** and **5b** and flavonol-based **7** (Figure 4). To note, the lack of cytotoxic effect for the unsubstituted coumarin (**5**, R = H) highlighted the role played by the 3-phenyl ring on the core structure, and the potency of **5b** indicated a positive influence of a fluorine compared to other substituents that led to inactive compounds. The reduction of the 2–3 double bond of flavone to give a flavanone core (**6**) seemed not to lead to any improvement in activity with respect to FAA. Coumarins **5a** and **5b** were also able to induce TNF-α and nitric oxide release.

### 2.2. DMXAA Analogues

**Figure 5:**
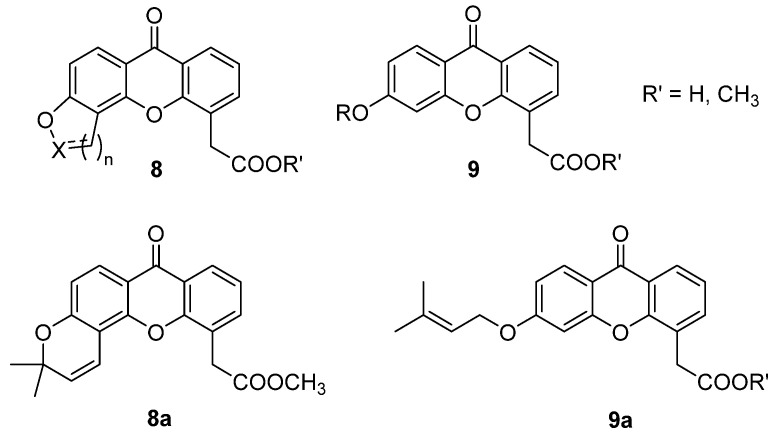
Cyclic DMXAA analogues and alkoxy derivatives.

Moving from FAA to XAA-analogues and considering the structure of the potent 5,6-DMXAA, different series of molecules were designed and synthetized, in which the substituents in positions 5 and 6 were included in different cyclic structures (**8**, Figure 5) to investigate structural requirements and steric limitations in this part of the molecule [21]. In particular, different easily synthetically accessible 5- or 6-membered oxygenated rings were introduced in the structure, since substitution with methoxy groups had proved to maintain high activity in XAA derivatives [22]. The synthetic intermediates (**9**, Figure 5), carrying bulky alkoxy groups in the favoured position 6 of the xanthone core, were tested together with the cyclic compounds and their esters for direct cytotoxicity on adenocarcinoma cell lines and for indirect toxicity, evaluating the increase in lytic properties for murine macrophages and human monocytes co-cultured with tumour cells.

Despite low direct toxicity, the compounds proved to induce an increase in the cytotoxic properties of murine macrophages comparable to or higher than the reference compound 5,6-DMXAA. Notably, most of them showed activity on human monocytes, and some proved to be more potent than the lead, the ester of the cyclic compound **8a** and both prenyloxy derivatives **9a** (Figure 5) being the most interesting derivatives. In addition, these compounds were able to induce TNF-α production in HPBMCs after 4 h of incubation.

**Figure 6:**
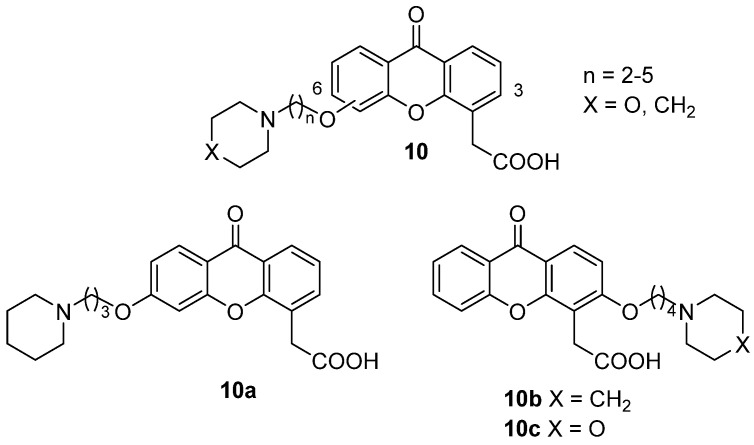
XAA analogues with basic side chains.

In a following paper, the xanthone scaffold was decorated in position 6 with saturated alkoxy chains of different lengths bearing a terminal amino moiety (piperidine or morpholine), to evaluate the effect of a basic function [23]. The same substitution pattern was also inserted in position 3 of the core structure, to obtain a more complete SAR picture. The compounds (**10**, Figure 6) were tested for direct cytotoxicity and for HPBMCs-mediated toxicity, and the induction of TNF-α and nitric oxide was evaluated.

Results of biological evaluation showed, apart from the expected low cytotoxicity, that the introduction of the selected basic side chain did not lead to an enhancement of the indirect antitumour effects of the new compounds on human monocytes but allowed in some cases the activity of the reference DMXAA to be retained, confirming that bulky substituents in the appropriate positions on the scaffold can be somewhat tolerated. In particular, **10a**, **10b** and **10c** (Figure 6) proved to be the most potent compounds. In addition, **10a**, being able to induce the release of TNF-α and NO, seemed to possess the same biological profile of DMXAA.

**Figure 7:**
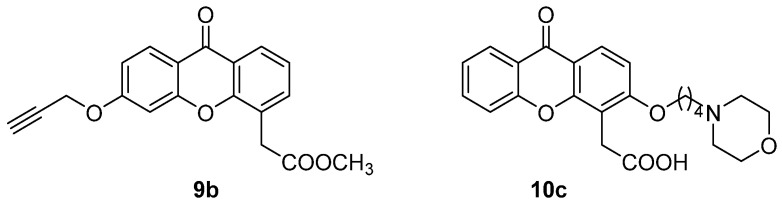
XAA derivatives selected for in vivo evaluation of antivascular properties.

Later, two XAA-analogues from the abovementioned series (**9b** and **10c**, Figure 7) were selected for further investigations to have a complete picture of their antitumour potential in comparison to the reference DMXAA [24]. For these molecules, the in vitro immune-modulating activity on a human monocyte cell line (MonoMac6 cells, MM6) and in vitro and in vivo antivascular properties were evaluated.

The selected compounds were shown to be able to induce immune modulation comparable to DMXAA; in particular, **10c** could stimulate TNF-α production to a higher extent. Notably, the release of TNF-α proved to be inversely dose-dependent, as seen in previous studies [25,26]. DMXAA proved to activate nuclear factor-kappa B (NF-κB) by inactivating its inhibitory factor IκB-α [27] through the allosteric interaction with the IKK kinase protein, triggering its phosphorylation and destruction. The selected compounds and especially **10c** significantly induced IκB-phosphorylation. The anti-vascular activity of the compounds was then studied in vitro by measuring the induction of apoptosis in human vein umbilical (HUVEC) cells and in vivo by evaluating growth inhibition of murine sarcoma F (SaF) cells. While DMXAA proved to be able to target and damage the tumour vascular network and to inhibit tumour progression, no such activity was seen for **9b** when tested at the same concentrations, and only low induction of apoptosis was observed for **10c**.

### 2.3. Miscellaneous

**Figure 8:**
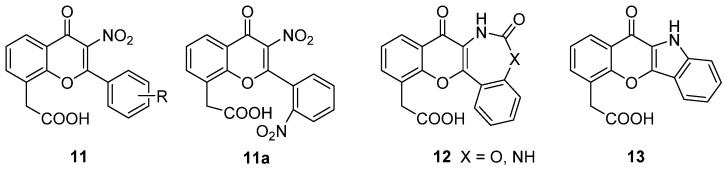
FAA analogues as inhibitors of aminopeptidase N.

In the same years, other research groups reported new flavonoid-based compounds as derivatives of FAA or XAA. In 2003, some analogues of FAA were designed and tested as inhibitors of aminopeptidase N (APN/CD13), overexpressed in some tumours, located in both human and murine endothelial vasculature but not in normal vessels. To this aim, 3-nitroFAA derivatives (**11**, Figure 8) bearing different substituents on the 2-phenyl ring (mainly nitro or methoxy groups), along with structurally constrained analogues (**12**–**13**, Figure 8) were designed and synthesized [28]. In particular, compound **11a** (Figure 8), carrying nitro groups in positions 3 and 2′, proved to efficiently and selectively inhibit APN/CD13, while modifications in the position of the substituent, its removal or replacement with a methoxy, led to reduction of the activity.

**Figure 9:**
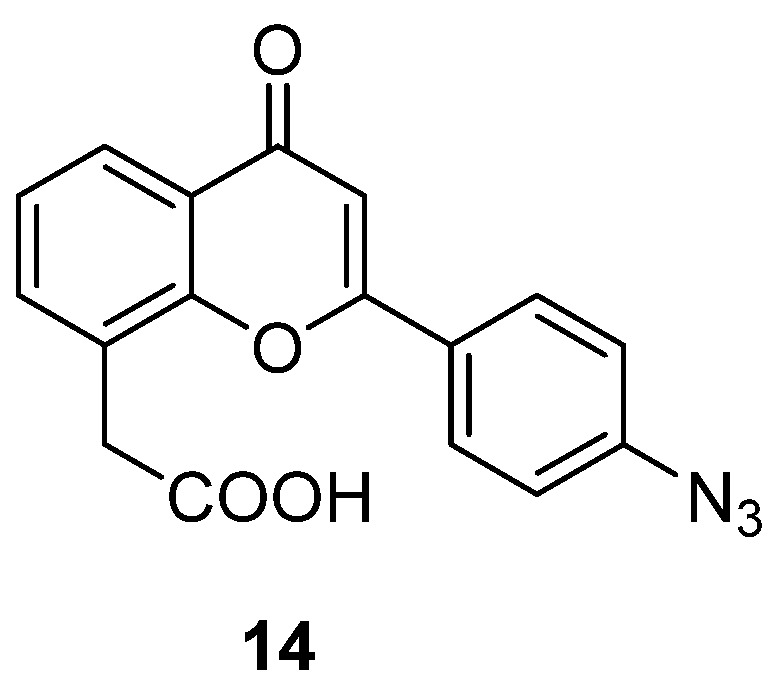
FAA analogue as affinity label.

In order to obtain more information about the mechanism of action of FAA and to identify its biological target, in 2005 a new analogue was designed, carrying an additional functional group that could react with a specific peptide tag as an affinity label, allowing the drug-bound protein to be located [29]. In detail, an azide moiety was introduced in the *para* position on the 2-phenyl ring giving compound **14** (Figure 9), which proved to maintain the biological activity observed with the parent compound and could be activated using the Staudinger reaction, by reacting it with a FLAG peptide–phosphine in mild conditions, similar to those used for standard biological assays.

**Figure 10:**
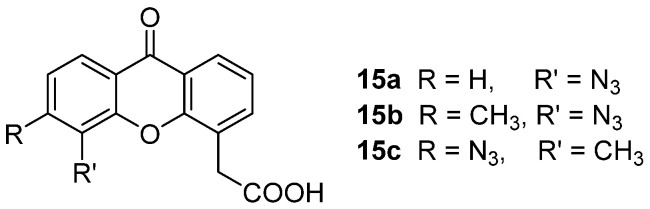
DMXAA analogues as photoaffinity labels.

The potential use of the azide moiety to develop analogues of the potent DMXAA as photoaffinity labels, that would covalently bind target proteins, was investigated by Ching and coworkers [30]. Here, this function was selected due to synthetic accessibility and lack of steric issues with respect to other photoreactive moieties, and it was introduced on the xanthone core in the most favourable positions for substitution, identified with previous SAR studies, giving compounds **15a**–**c** (Figure 10). While **15c** proved to be chemically unstable and was not studied further, the activities of **15a** and **15b** were evaluated, and both were shown to retain the biological profile of the parent compound, although more toxic and less potent. Moreover, **15b** proved to undergo photoreaction to specifically bind to proteins extracted from murine splenocytes.

## 3. Targeted SAR Studies 2013–2021

In the first twenty years following DMXAA discovery, despite extensive medicinal chemistry efforts, no significant improvement of its biological profile nor better comprehension of the molecular basis of its mechanism of action were achieved. In 2012, the discovery of the possible involvement of the STING pathway in DMXAA anticancer action paved the way for a renaissance of the studies on this molecule [13]. STING is a transmembrane protein involved in sustaining innate immune responses in the tumour microenvironment. The formation of cytosolic chromatin fragments is often related to malignant transformation, and DNA leakage in cancer cells cytosol may occur, triggering the innate immunity response; the cyclic GMP–AMP synthase-stimulator of interferon genes (cGAS-STING) pathway gets activated, leading to the expression of type I interferon (IFN) in cancer cells, initiating innate anti-cancer immunity. The acknowledged role of cGAS-STING pathway in sustaining anti-tumour innate and adaptive immunity makes its pharmacological activation a valuable strategy to be exploited for anticancer therapy [31]. The ability of DMXAA to directly bind STING was proved in multiple murine models and was associated with a consistent tumour regression. Unfortunately, it was found that the affinity of this drug for STING was limited to murine protein (mSTING), since in human cells this interaction was unable to induce type I IFN expression [14]. Despite these disappointing results, probably due to STING polymorphism, the possibility to focus research on a specific target has led to a revival of studies on DMXAA, its structure and its derivatives. Therefore, different research groups returned to the flavonoid-related structures of FAA and XAA to develop new analogues, for which both antivascular and immunomodulating properties were evaluated.

### 3.1. Recently Developed FAA Derivatives

**Figure 11:**
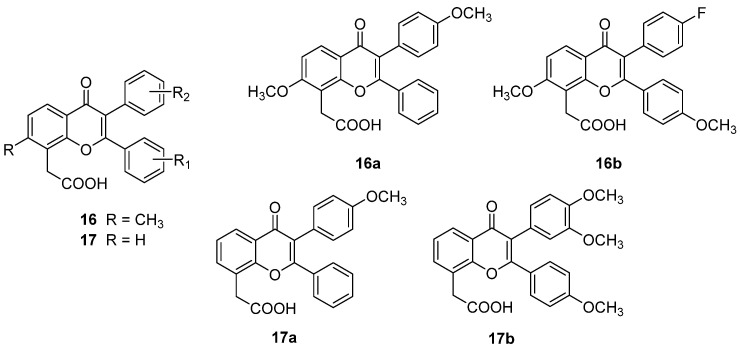
2,3-Diarylchromones as FAA derivatives.

In 2014, a novel series of FAA derivatives was synthesized bearing a 2,3-diarylchromone scaffold (**16**, Figure 11), designed by considering the structures and antitumour activities of literature analogues and known isoflavones [32]. The new derivatives were characterized by a methoxy group in position 7 and methoxy- or halogen-substituents on the benzene rings. The compounds showed moderate cytotoxicity and, more interesting, **16a** and **16b** (Figure 11) were endowed with indirect toxicity on A549 lung adenocarcinoma cells that proved to be higher when cells were co-cultured with HPBMCs than with murine macrophages, in line with the reference DMXAA. In addition, **16a** was able to induce TNF-α production to a higher extent with respect to DMXAA.

Later, the same research group synthesized another series of **16**-based analogues, in which the 7-methoxy group was removed (**17**, Figure 11), which was more potent than the previous ones [33]. In particular, while compounds carrying electron withdrawing groups on the phenyl rings showed moderate direct cytotoxic activity, methoxy-substituted analogues proved to be endowed with indirect toxicity comparable or higher than the reference DMXAA and to induce TNF-α production, **17a** and **17b** (Figure 11) being the most interesting compounds.

**Figure 12:**
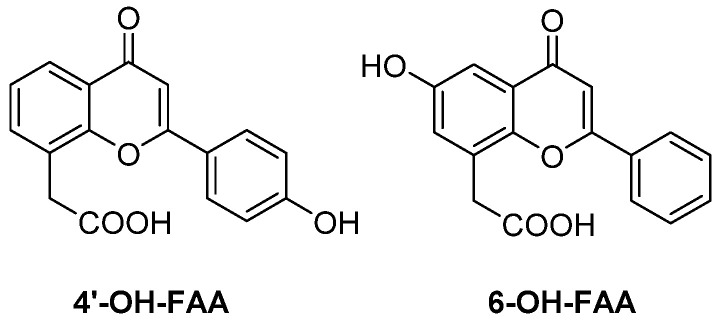
Representative FAA metabolites in murine cells.

FAA is known to be metabolized by murine microsomes to give different hydroxy- and epoxy-derivatives, but it undergoes very low metabolism in human microsomes. Recently, the two main metabolites, 4′-OH-FAA and 6-OH-FAA (Figure 12), extensively found in mice but not in humans, were tested for their antivascular effects on EA.hy926 cells, derived from the fusion of human umbilical vein endothelial cells with the permanent human A549 cell line [34]. Notably, 6-OH-FAA was able to induce morphological changes at significantly lower concentrations with respect to 4′-OH-FAA and FAA itself, and to disrupt preformed capillary networks. In addition, it inhibited microtubules formation by activating the RhoA/Rho signalling pathway. Based on these findings, it was suggested that the potent antivascular activity of FAA in mice, and the lack of activity in human tumour cells, could be mediated by its metabolite 6-OH-FAA.

### 3.2. Recently Developed DMXAA Derivatives

**Figure 13:**
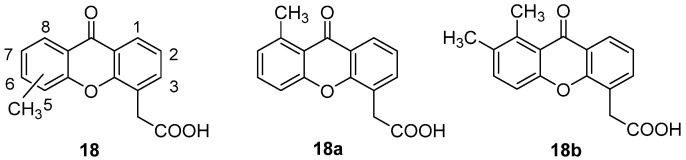
Methyl-substituted DMXAA analogues.

To gain more insight into the peculiar selectivity for murine tumour cells and the consequent lack of antitumour effect on human cells, demonstrated by DMXAA and with the aim of identifying effective human antitumour agents, the previously synthesized analogues carrying methyl groups in different positions on the xanthone scaffold (**18**, Figure 13) were considered, in 2013, by Ching and coworkers [35], and the pattern of cytokine induction and the anti-vascular effects were evaluated in both human and murine leukocytes.

Results clearly indicated distinct biological effects for the different monomethyl derivatives, since substitution in positions 3, 5 and 6 led to compounds with significant activity on murine cells, as the disubstituted 5,6-DMXAA, whereas 2-, 7- and 8-methylXAA showed remarkable selectivity for human cells and proved to be inactive in the murine model. In particular, 8-methylXAA (**18a**, Figure 13) and the disubstituted 7,8-DMXAA (**18b**, Figure 13) proved to efficiently induce a panel of cytokines (IL-6 and IL-8) in human leukocytes as well as a significant antivascular effect, through inhibition of tube formation. Notably, the lack of biological effects seen in murine systems for these compounds called attention to the need for appropriate models for a more appropriate evaluation of the potential antitumour activity of this class of molecules. Considering the very recent identification of STING as a potential molecular target of DMXAA, it was also suggested that differences in murine and human protein could be responsible for the peculiar species specificities observed in the SAR of these XAA derivatives.

**Figure 14:**
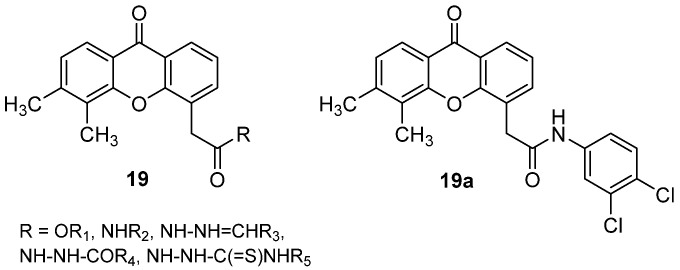
Carboxylic acid derivatives of DMXAA.

Different series of xanthone analogues were also synthesized, in which the carboxylic acid was modified to obtain esters, amides, arylidene hydrazides, diacylhydrazides and acyl thiosemicarbazides (**19**, Figure 14) [36]. The compounds were tested for both direct cytotoxicity against a panel of tumour cells and antiangiogenic properties by evaluating tube formation and migration in human vein umbilical (HUVEC) cells. Among these derivatives, some amides and arylidene hydrazides showed cytotoxic activity in the micromolar range and, remarkably, were proven to induce morphological changes in HUVEC cells and reduce cell migration (whereas no such effect was shown by the reference DMXAA). In particular, **19a** (Figure 14) emerged as the most interesting compound and was the subject of docking studies that suggested its potential interaction with VEGFR2 and EGFR.

**Figure 15:**
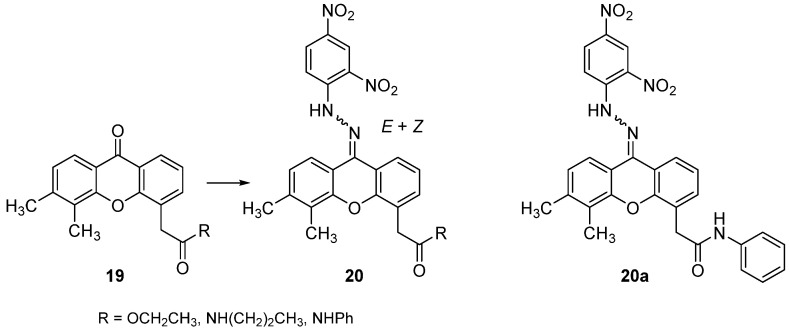
DMXAA hydrazone derivatives.

The same research group synthesized three DMXAA ester or amide derivatives, in which the carbonyl function in position 9 was removed via the formation of a 2,4-dinitrophenylhydrazone function (**20**, Figure 15) [37]. For the new compounds (together with their xanthone counterpart) only preliminary cytotoxicity studies on different cell lines were performed, suggesting activity in the micromolar range for the two xanthone amides and for the hydrazone **20a** (Figure 15) in some of the tested cell lines.

**Figure 16:**
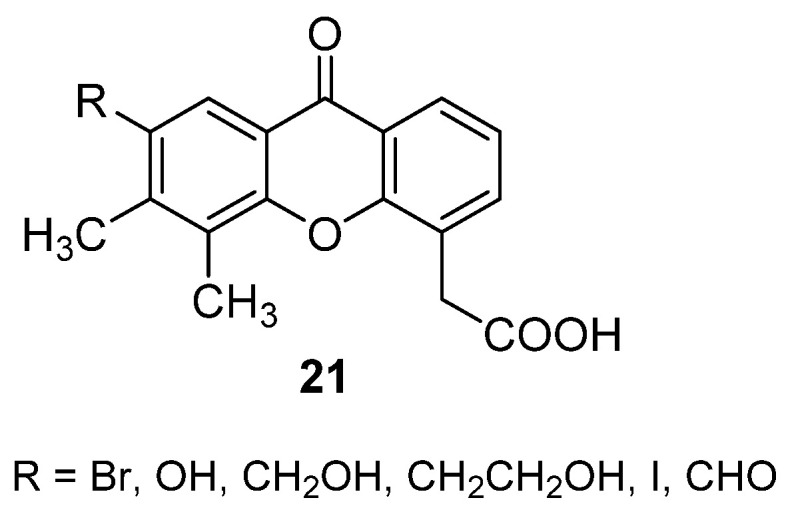
7-Substituted DMXAA analogues.

Considering the identification of mSTING as the molecular target of DMXAA and the evidence of species specificity, new analogues carrying different H-bond acceptor or donor groups in position 7 of the xanthone core were recently designed (**21**, Figure 16) based on the X-ray crystal structure of DMXAA bound to a mutated human STING (hSTING) that suggested a potential additional H-bond interaction for 7-substituted derivatives [38]. The compounds and their ester synthetic intermediates were tested to assess their binding to both the murine and human proteins and, while bromo- and hydroxy-xanthones showed promising affinity for mSTING, none of the synthesized molecules proved to efficiently bind to the human counterpart.

### 3.3. DMXAA Combinations/Hybrids

**Figure 17:**
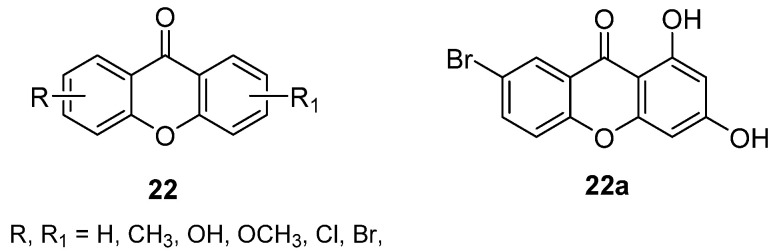
Novel antitumour xanthones for DMXAA combinations.

Recently, DMXAA was studied as part of a drug combination involving variously decorated xanthones (**22**, Figure 17), aimed at evaluating the possible synergistic effects of the two structurally-related molecules [39]. The synthesized xanthones were tested for direct cytotoxicity toward different human tumour cell lines, and only the 1,3-dihydroxy substitution pattern, together with electron withdrawing groups, seemed to grant activity in the low micromolar range, as any modification proved to be detrimental. The most potent compounds were then tested in combination with DMXAA (1:1 molar ratio), which proved to enhance their cytotoxicity, especially for compound **22a** (Figure 17) on MDA-MB-231 cells. Furthermore, for this compound and its combination with DMXAA, the induction of apoptosis and the regulation of p53/MDM2 were observed.

**Figure 18:**
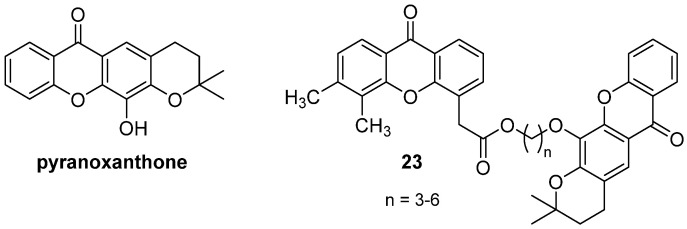
Pyranoxanthone and hybrid derivatives.

In a following study, the combination of DMXAA and pyranoxanthone (Figure 18), a novel xanthone-based inhibitor of p53-MDM2 interaction, was evaluated, and four hybrid molecules were synthesized by coupling the two synthons through an alkyl tether (**23**, Figure 18) [40]. Both the combination of the two molecules and the hybrids showed higher cytotoxic activity than the single agents, particularly on MCF-7 and HepG-2 cells, the hybrids with longer linker chains being the most interesting compounds, with activities in the submicromolar range. Moreover, the hybrid with a 6-carbon tether proved to induce apoptosis and to regulate p53/MDM2 to a higher extent with respect to the single synthons and the combination.

The vascular shutdown caused by treatment with VDAs such as DMXAA has been shown to be associated with the establishment of hypoxic and necrotic areas in the tumour, which would induce the activation of HIF-1α protein, subsequently leading to the development of new blood vessels and immunosuppression, promoting tumour regrowth. Thus, the potential synergistic effect that could be obtained with the combination of DMXAA and digoxin, a known inhibitor of HIF-1α expression [41], was recently considered [42]. The administration of these agents in B16-F10 murine melanoma showed remarkable reduction of tumour blood vessels and enhanced induction of immune cells, together with a reduction of the expression of HIF-1α, increasing treatment efficacy.

## 4. Conclusions and Future Directions

Cancer treatment undoubtedly represents one of the greatest challenges for medicinal chemistry and pharmacology, and exploring new therapeutic strategies and targets is always a valuable research approach. Angiogenesis is essential for tumour progression and metastases development, and VDAs are a peculiar class of anticancer compounds aimed at disrupting established tumour vasculature. A number of NPs were found to act as VDAs and constitute a source of structural templates to be properly modified in order to increase the therapeutic potential and combine different anticancer mechanisms. The synthetic flavone-based FAA and its derivatives, mostly DMXAA and other xanthone-related compounds, seemed to have achieved this goal, but finally gave disappointing results in clinical trials, revealing a species-specificity in their mechanism of action that made them ineffective in humans. Despite a number of chemical modifications that expanded the SAR of these scaffolds and the ability of some derivatives to target human cells, no significant improvement was observed up to 2012, when the discovery of STING as a possible distinctive target for DMXAA gave a second chance to these structures. New encouraging medicinal chemistry studies are now underway, also taking advantage of a more focused target selection to evaluate both antivascular effects and immunomodulation. Indeed, promising VDA candidates were recently identified, worthy of further evaluation.

Furthermore, several beneficial features of DMXAA must be taken into account, in particular its low toxicity and well-characterized and predictable pharmacokinetics. Moreover, this compound showed a good potential for combination therapy with a wide number of antitumour drugs, acting with different molecular mechanisms. Considering future medicinal chemistry perspectives, this allows to keep the spotlight on this structural core, aiming at performing further modifications to obtain more effective new analogues and multipotent ligands, able to engage different targets involved in cancer. The multitarget approach should, however, be carefully considered, as it can indeed allow to improve the compliance of a patient that is often overloaded by different drugs, but it can reasonably lead to the development of molecules endowed with worse pharmacokinetic properties with respect to the single entities. On the other hand, the identification of a potential target and the related latest findings could bring back attention to previously reported FAA and XAA derivatives that could undergo further investigations. More focused biological evaluation could open the way for the design and synthesis of novel libraries of variously decorated compounds, leading to a better definition of the structural requirements to obtain human tumour active VDAs. A suitable model for in vivo testing appears then as an imperative requirement to exploit this huge potential.
